# High helminthic co-infection in tuberculosis patients with undernutritional status in northeastern Ethiopia

**DOI:** 10.1186/s40249-019-0600-2

**Published:** 2019-10-18

**Authors:** Fikru Gashaw, Samuel Bekele, Yalemtsehay Mekonnen, Girmay Medhin, Gobena Ameni, Berhanu Erko

**Affiliations:** 1 Aklilu Lemma Institute of Pathobiology, Addis Ababa University, Addis Ababa, Ethiopia; 2 Department of Microbial, Cellular and Molecular Biology, College of Natural Sciences, Addis Ababa University, Addis Ababa, Ethiopia; 3 Department of Biology, College of Natural and Computational Sciences, Kotebe Metropolitan University, Addis Ababa, Ethiopia

**Keywords:** Tuberculosis, Parasitosis, Co-infection, Undernutrition, Northeastern Ethiopia

## Abstract

**Background:**

Tuberculosis and parasitosis are the widely distributed diseases in Ethiopia with the leading cause of mortality and morbidity, respectively. There has been no information on the status of co-infections of tuberculosis and parasitosis in Oromia Zone of Amhara Region and South Wollo, Ethiopia. Hence, this study primarily focuses on determining the status of tuberculosis and parasitosis co-infections and associated factors.

**Methods:**

The study was conducted in Oromia Special Zone of the Amhara Regional State and South Wollo Zone, northeastern Ethiopia from April 2015 to January 2017. Tuberculosis cases confirmed by health personnel at the health institutions were the source of the study sample. In a cross-sectional study 384 smear positive pulmonary and extra-pulmonary tuberculosis cases were recruited. Faecal specimens provided by the study participants were examined for parasitic co-infections using direct saline microscopic test, Kato-Katz and concentration techniques. Nutritional status was determined using body mass index and mid-upper arm circumferences. Data were analyzed using descriptive statistical methods and Pearson chi-square.

**Results:**

Tuberculosis and parasitosis co-infection prevalence was 10.8%, and the proportion of intestinal helminths accounted for 9.7% while intestinal protozoa accounted for 1.9%. Cases with single parasitic infection was 89.3% among co-infected individuals. Co-infection of both disease was not significantly associated with gender and age (P > 0.05). The prevalence of undernutrition was 58.6% as determined using body mass index and 73.0% as determined using mid-upper arm circumference with no significant association with gender. Among all forms of tuberculosis cases (384) screened for the study, the bacterial positivity was relatively more common in males (55.5%) than females (44.5%). Tuberculosis lymphadenitis was found to be the most prevalent (85.9%) form of extra-pulmonary tuberculosis with cervical adenopathy (75.3%) being the commonly existing disease.

**Conclusions:**

The rate of helminthic co-infection is predominantly high than that of intestinal protozoa. Single parasitic co-infection was more common than double or multiple co-infections. Both body mass index and mid-upper arm circumference anthropometric parameters revealed greater risk of undernutrition in tuberculosis patients. Thus, screening and prompt treatment of parasites in tuberculosis patients and a support of nutritional supplementation for malnourished tuberculosis patients should be further studied which might enhance the disease treatment and minimize the risk of its complexity.

## Multilingual abstracts

Please see Additional file 1 for translations of the abstract into the five official working languages of the United Nations.

## Background

Tuberculosis (TB) is the leading bacterial infectious diseases that cause profound mortality and morbidity throughout the world. It is a highly chronic unpardonable respiratory disease mainly affecting lungs and other body parts as extra-pulmonary tuberculosis (EPTB). The disease is primarily caused by *Mycobacterium tuberculosis* (*Mtb*) having an infection rate of over one-third of the global population [1]. Other members of the *Mtb* complex like *M. bovis*, *M. africanum* and *M. microti* can also cause human TB in rare cases [2]. In 2017, TB caused an estimated death of 1.6 million of which 0.3 million are those co-infected with HIV and the disease continuous as one of the top ten leading causes of mortality. Although there are some progress in Ethiopia in terms of reducing incident rate of TB, the country still remains as one of the top 30 high TB burden countries [3].

It is also estimated that 3.5 billion of the global population are infected with intestinal parasites of whom 450 million are ill as a result of the infection [4]. These parasites are heterogeneous group of protozoa and helminths that live part or all of their lives in the hosts where they derive necessary nutrients, grow, and reproduce [5, 6]. The parasites interfere with nutritional and immune profiles of an individual promoting secondary infections, such as *Mtb*. Prevalence of intestinal parasitic infection is remarkably high in sub-Saharan African countries where TB is also common [5, 7–10].

Helminth infections can reactivate latent TB and aggravate the disease expression [11]. Limited epidemiological studies in Ethiopia have shown that chronic parasitic infection could increase the risk of TB [12–14]. Other conditions like *Diabetes mellitus*, malnutrition, and malignancies are also known to enhance the risk of TB infection [15, 16].

Identifying nutritional status of an individual could bring a substantial role in the prevention and control of the diseases. The relative risk of TB infection among persons in the lowest body mass index (BMI) category was more than five-fold higher compared to the group that fall in the highest BMI category [17]. Undernutrition affects cell-mediated immunity which is vital for the principal host defense mechanism against TB. The likelihood of developing primary or latent TB infection to active disease in undernourished group is greater [18]. This evidence supports the recommendation that patients with TB should be nutritionally assessed and get nutritional care and support which directs nutrition screening, assessment and management as an integral component of TB treatment and care [19].

As far as this study is concerned, parasitic co-infection and nutritional status of tuberculosis patients are not known in the study area. Thus, the study was primarily undertaken to determine the prevalence of parasitic co-infection and malnutrition among TB cases in Oromia Zone of Amhara Region, and South Wollo Zone. The findings are expected to contribute towards proper treatment and management of TB in the study area and beyond.

## Methods

### Description of the study area

The study was conducted in Oromia Special Zone of the Amhara Regional State and South Wollo Zone, northeastern Ethiopia. The area was selected purposely for this study that there was no any reported documents that shows the status of TB-parasitic co-infections and the associated factors. Both zones have administrative centers in Kemise and Dessie Towns. The former Town is the administrative center for the Oromia Special Zone since 1994 with a latitude of 10°43′27.4“N and longitude of 39°52’24.03”E. The town is found at an altitude of 1446.88 m and a distance of 325 km northeast of Addis Ababa. On the other hand, Dessie Town is the capital of South Wollo Zone having a north latitude and east longitude of 11°8′ and 39°38′, respectively. Dessie has an average altitude of 2475.1 m and located 401 km northeast of Addis Ababa [20].

**Fig. 1:**
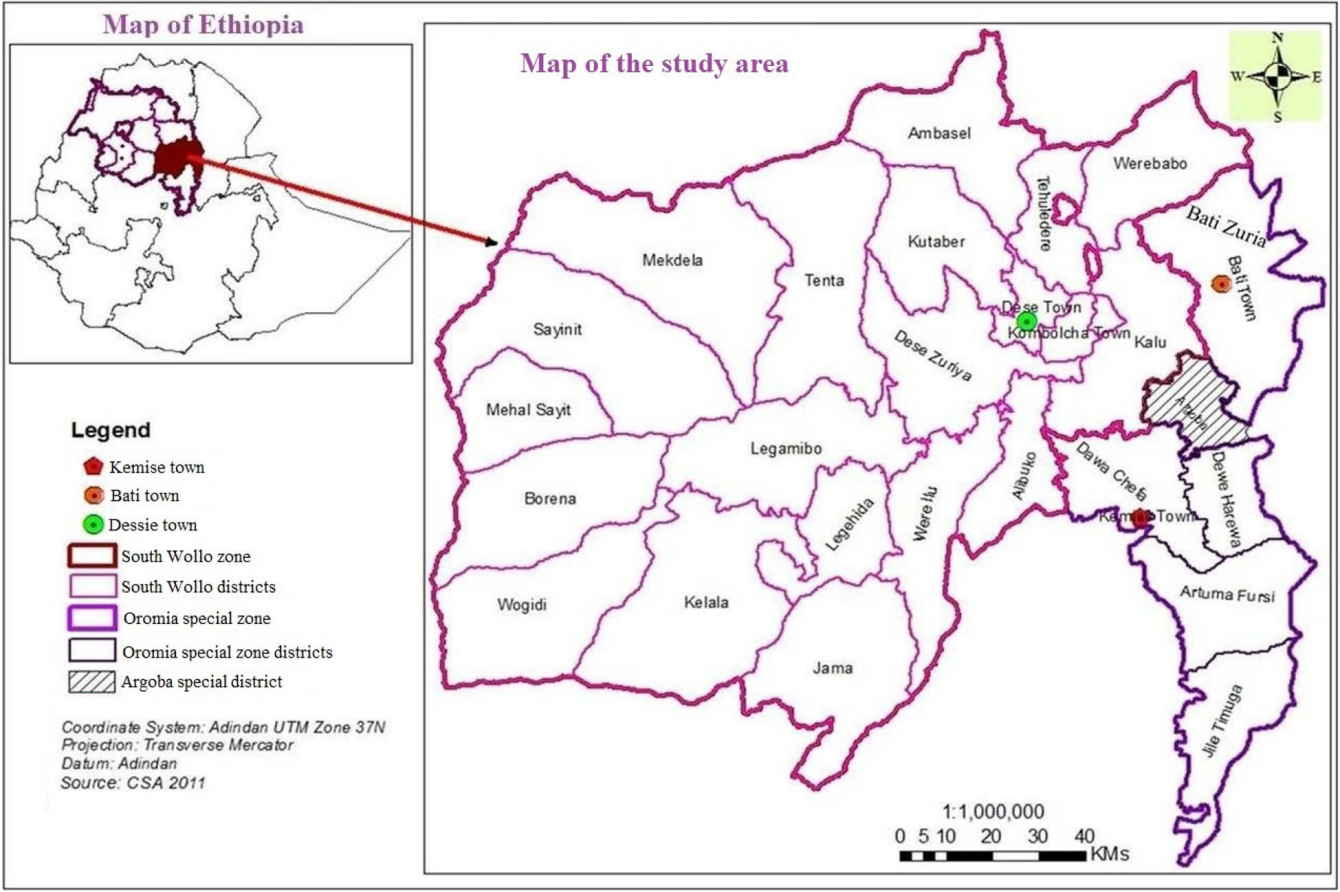
Map of Ethiopia showing the catchment areas of the patients, April 2015 to January 2017

Administratively, the Oromia Special Zone has seven districts. Residents of the zone receive governmental health service from 27 health centers and one general hospital that partially began its function at Kemise Town in 2015. The two towns administration of Oromia Special Zone (Kemise and Bati) have one governmental health center each and were accessible for our pulmonary tuberculosis (PTB) sample collection. Similarly, South Wollo Zone has 18 districts and two town administration (Dessie and Kombolcha) (Fig. 1).

### Study population

TB cases confirmed by the health personnel (pathologist, medical doctors, health officers, nurses and laboratory technologists) and those who fulfilled the inclusion criteria were included in the study. The samples (sputum, Fine-needle aspirates (FNAs) and stools) were collected on the spot from consenting participants until the expected sample size was achieved.

In addition, non-active TB cases using clinical symptoms (cough, sputum production, haemoptysis, dyspnea, anorexia, chest pain, fever, drenching night sweat, weight loss and weakness), apparently healthy individuals matched with TB cases by age (with a difference of + 5 year), sex and place of living were used as control to compare the nutritional status. The controls were not bacteriologically and parasitologically confirmed for TB and parasitosis, respectively. On the other hand, those individuals who came to the health facilities with the TB cases but didn’t fulfill the indicated criteria were excluded.

All TB cases from April 2015 to January 2017 who were 18 years and older and willing to participate in the study based on their written voluntary consent were included. This is due to small proportion of TB cases younger than 18 years. Those with severe TB and unable to provide sputum, and those who gave their sputum for TB examination but couldn’t provide faecal specimens were excluded from the study.

Preliminary survey was made for 3 months in all governmental health facilities of the seven districts in Oromia Special Zone. Based on the availability of samples (convenience sampling) and transportation access, the data were collected from Kemise and Bati Town health centers of the Special Zone. In addition, to get more TB cases Dessie Referral Hospital (DRH), Bikat Higher Diagnostic Laboratories (BHDL), Dessie Health Center (DHC) and Boru Meda Hospital (BMH) were also the sample collection sites from Dessie Town (Fig. 1). Clinically suspected and bacteriologically confirmed patients using sputum smear test in all study sites were included in the study. GeneXpert was additionally done for TB diagnosis at DRH. For EPTB, the suspected samples were collected and examined at BHDL by experienced pathologist.

### Sample size estimation

Institution-based cross-sectional study design was performed. For determination of sample size, co-infection prevalence (33.3%) of *Mtb* and intestinal parasitic infections (IPIs) in northwest Ethiopia [13], 95% confidence in the estimate and 5% margin of error were considered. This resulted in a sample size of 341 smear positive TB cases. To compensate for the non stool providing TB cases, inadequate sputum specimen for culture and to have better coverage of the study population an additional 12.6% of the minimum sample size was considered and the final sample size was calculated as 384.

### Sample collection and processing

Dry, translucent, leak-proof 50 ml capacities of falcon tubes were used to collect a minimum of 3–5 ml sputum sample from BMH where drug resistant TB cases were handled and treated. Similarly, a 30 ml sputum cup was used for sputum collection from the rest of the study sites. The data were safely recorded on the spot of sample collection to determine the patients’ and control group socio-demographic characteristics.

Direct microscopic examination of the sputum was done by fluorescent microscope (FM) at a magnification of 200×. The bacterial grading scale was considered as negative when no AFB (acid fast bacillus)/in at least 30 fields, scanty (1–29 AFB/30 fields), 1+ (30–299 AFB/30 fields), 2++ (10–99 AFB/ in at least 15 fields) and 3 +++ (> 100 AFB/field in at least 6 fields). Positive specimens from patients were kept at a range of − 10 to − 20 °C refrigerator until being transported to Aklilu Lemma Institute of Pathobiology (ALIPB) for culturing following the protocol described elsewhere [21].

FNA samples from patients suspected for EPTB was collected using a 21-gauge needle attached to a 10 ml syringe with maximum care and safety by an experienced pathologist [22]. Afterwards, the Ziehl-Neelsen smear technique was performed by the same pathologist to check its positivity. From positive participants of the smear, a suction of about 1 ml samples were collected for this research purpose and preserved in sterile and tightly closed nunc tubes with phosphate buffer saline of 1 ml at pH 7.2 and kept in the same refrigerator [23, 24]. Finally, both sputum and FNA samples were transported from temporary storage site of the health institutions in a cold chain of 4 °C to TB laboratory of ALIPB in Addis Ababa. At ALIPB the samples were kept in a deep freezer of – 80°C until culturing was done [25].

### Lowenstein-Jensen (LJ) media preparation and mycobacterial culturing

A stock of selective LJ media was used for the preparation of *Mycobacterium* culture based on the manufacturer’s instruction (Fluka Chemie GmbH, Switzerland) and the standard operating procedure of TB laboratory at ALIPB. Glycerol (0.75%) and pyruvate (0.6%) was added to enrich the media for the cultivation of *Mtb* and *M. bovis*, respectively. The prepared media were left for 48 h to check for contamination before culturing the bacteria. Poor quality media were also identified and discarded. All the inoculated LJ slants in culture tubes were incubated aerobically at 37 °C in a slanted position and contamination was checked daily for the first week. The inoculated media were placed in an upright position starting from the second week and colony formation was checked for eight consecutive weeks. For weakly grown colonies, sub-culturing of the colony was done in another new medium [26].

### Parasitological examination

Faecal specimens were collected using stool cup with a tight fitting lid and two applicator sticks given to the TB patients to bring a sizable stool sample of his/her own. The slides were labeled with identification number and a drop of saline (0.9%) was placed at the middle for direct wet mount faecal examination. A small proportion (about a match stick size) of the specimen was picked up and mixed with the saline and covered with cover slip for entire microscopic observation under low power objective. A single Kato-Katz thick smear per stool sample was also prepared from the stool using a template delivering 41.7 mg of feces. A multiplication factor of 24 was used to convert egg count into eggs per gram of stool [27]. A portion of the fresh stool samples (about 1 g) preserved in 10 ml sodium acetate-acetic acid-formalin (SAF) solution was transported to Medical Parasitology Laboratory of Aklilu Lemma Institute of Pathobiology and processed using saline ether concentration techniques for microscopic examination. Lack of repeated stool examination for parasite test in this study was considered as a limitation of the study.

### Anthropometric measurements

Age, sex, height, weight and mid-upper arm circumference (MUAC) were measured and recorded. The BMI of participants was calculated from individuals’ weight in kilograms and height measured in meters to one decimal place using calibrated measurements. In BHDL, measurements for BMI were also done using calibrated ultrasonic height and weight measuring machine. The print outs of the machine were collected and registered for each of the participants. The result was categorized as underweight (BMI ≤ 18.5 kg/m^2^), normal (18.5 kg/m^2^ < BMI ≤ 25 kg/m^2^), overweight (25 kg/m^2^ < BMI ≤ 30 kg/m^2^), and obese (BMI > 30 kg/m^2^). Weighing of the participants was made to the nearest 0.1 kg and the proportion of mildly (BMI 17.0–18.5 kg/m^2^), moderately (16.0 kg/m^2^–16.9 kg/m^2^) and severely (BMI < 16.0 kg/m^2^) undernourished participants identified [28, 29]. Mid-upper arm circumference was measured to the nearest 0.1 cm and also used for the assessment of malnutrition. Considering the cutoff values for MUAC, the study participants were categorized as undernourished when MUAC is ≤23 cm for males and ≤ 22 cm for females [30, 31].

### Quality control

Questionnaire was prepared in English and then translated into the local languages (Amharic and Afaan Oromo) by an expert who was fluent in both languages to maintain its consistency. Training was given for data collectors and supervisors. Pre-testing of the questionnaire was made on 30 study participants for its clarity and appropriateness which was latter included in the study. Data collection process was strictly followed day to day by the supervisor and principal investigator. In addition, quality of reagents and instruments was checked by experienced laboratory technicians using negative and positive control slides. Each of the specimens was done by two experienced laboratory technicians. In cases where the results were discordant, a third reader was used whose result was considered as the final result. Continuous quality control of the health institutes was also made by Dessie regional laboratory experts as an external quality assurance. In addition to the trainings on BMI measurements, the instruments were also calibrated for the research work.

### Data management and analysis

The recorded data was checked for completeness and consistency, and then entered into Microsoft Excel 2007 spreadsheets. This data was then exported to IBM SPSS Statistics for Windows, Version 25.0. (Armonk, NY: IBM Corp., USA) program for analysis. Percentage and mean were used to descriptively summarize the data. Association of TB-parasitosis co-infection and malnutrition with gender and age was analyzed using chi-square. Differences were considered significant at a *P*-value of < 0.05.

## Results

### Socio-demographic characteristics and tuberculosis infections

A total of 384 TB cases (213 males and 171 females) were involved in the study. Most of the cases were recruited from South Wollo Zone (*n* = 247). Their age ranged from 18 to 75 years with a mean age of 33.7 (SD = 12) having no significant difference between males (mean = 34.5, SD = 12.3) and females (mean = 32.7, SD = 11.4). The average household size of the TB cases was 4.4. Pulmonary TB cases accounted for 74.5% (286/384), the overall prevalence of TB was highest (67.0%) in the 18–37 years age group and those with family size ranging from 3 to 5 were more affected (Table 1).

**Table 1:** Background characteristics of tuberculosis patients recruited from health institutes of Oromia Special Zone and South Wollo Zone, northeastern Ethiopia, April 2015 to January 2017

Characteristics	Number	Percent
Sex (*n* = 384)		
Male	213	55.5%
Female	171	44.5%
Age in years (*n* = 371)		
18–27	133	35.8%
28–37	116	31.3%
38–47	68	18.3%
≥48	54	14.6%
Marital status (*n* = 333)		
Single	116	34.8%
Married	185	55.6%
Divorced	32	9.6%
Family size (*n* = 323)		
< 3	65	20.1%
3–5	161	49.9%
≥6	97	30.0%
Occupation (*n* = 345)		
Government employed	29	8.4%
Merchant	57	16.5%
Farmer	138	40.0%
Daily laborer	31	9.0%
No job/unemployed	40	11.6%
Student	26	7.5%
Others ^a^	24	7.0%
Religion (*n* = 348)		
Christian	119	34.2%
Muslim	229	65.8%
Ethnicity (*n* = 346)		
Amhara	295	85.3%
Oromo	46	13.3%
Others^b^	5	1.4%
Educational status (*n* = 347)		
Illiterate (unable to read and write)	154	44.4%
Primary (Grade 1–8)	99	28.5%
Secondary (Grade 9–12)	61	17.6%
Above secondary (Grade > 12)	33	9.5%

^a^Housewife/catering/pensioner/casher, ^b^ Tigre/Afar

### *Mycobacterium tuberculosis* -parasite co-infections

**Fig. 2:**
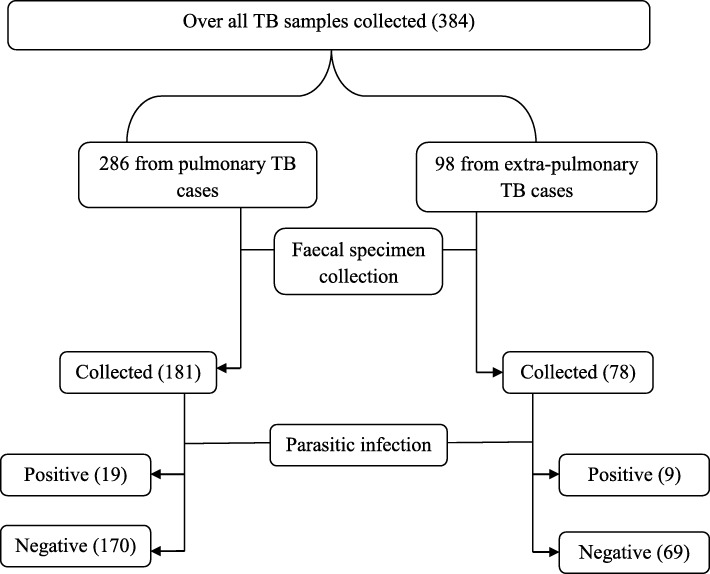
A flowchart outlining the general procedure of sample collection and TB-parasitic co-infections

A total of 259 faecal samples (142 males and 117 females) were collected and examined. The remaining tuberculosis cases refused to give faecal specimens and some of them were also severely sick and did not provide specimen. From the study, comparable intestinal parasitic infection was found among pulmonary TB cases (10.5%, 19/181) and extra-pulmonary ones (11.5%, 9/78) (Fig. 2).

The overall *Mtb*-parasite co-infection was 10.8% (28/259). From the total co-infected cases, 89.3% (25/28) had single parasitic infection, while 7.1% (2/28) had double infection, and 3.6% (1/28) had four infections. The infection of intestinal helminths accounted for 9.7% and those of intestinal protozoa accounted for 1.9%. Although there was greater helminthic co-infection than protozoa co-infection among tuberculosis patients in the study area, the difference was not statistically significant (*χ*^2^ = 6.000, df = 4 and *P* = 0.199).

*Schistosoma mansoni* infection was the most prevalent parasitic co-infection (4.25%), followed by *Ascaris lumbricoides* (2.32%)*. Trichuris trichiura*, *Enterobius vermicularis* and *Hymenolepis nana* were the other helminthic co-infections whereas *Entamoeba histolytica/dispar* and *Giardia lamblia* cysts were the only detected protozoan parasitic co-infections (Table 2).

**Table 2:** Parasite species among tuberculosis cases in Oromia Special Zone and South Wollo, northeastern Ethiopia, April 2015 to January 2017

Types of parasites	Number (percentage) of infection
*Schistosoma mansoni*	11 (4.3%)
*Ascaris lumbericoides*	6 (2.3%)
*Trichuris trichiura*	2 (0.8%)
*Enterobius vermicularies*	2 (0.8%)
*Giardia lamblia*	2 (0.8%)
*Entamoeba histolytica/dispar*	1 (0.4%)
Hookworm	1 (0.4%)
*Schistosoma mansoni* and *Giardia lamblia*	1 (0.4%)
*Ascaris lumbericoides* and *Enterobius vermicularies*	1 (0.4%)
*Schistosoma mansoni*, *Hymenolepis nana, Giardia lamblia* and hookworm	1 (0.4%)
Total	28 (10.8%)

The association of TB-parasite co-infection between males (60.7%, 17/28) and females (39.3%, 11/28) was not statistically significant (*χ*^2^ = 439, df = 1 and *P* = 0.507). Similarly, significant difference of *Mtb*-parasitic co-infection was also not observed across different age groups (*χ*^2^ = 36.238, df = 40 and *P* = 0.640).

### Assessment of nutritional status

The overall mean BMI of TB cases was 18.2 kg/m^2^. Among the study participants (50.0%, 192/384) had chronic energy deficiency, 34.6% (133/384) were normal and data was missing for 15.4% (59/384). Of the chronic energy deficient TB cases, 61.5% (118/192) were mildly undernourished, 21.9% (42/192) were moderately undernourished and 16.7% (32/192) were severely undernourished.

Of 237 TB cases whose faecal specimens were examined, 58.6% (*n* = 139) were underweight and there was no significant difference (*P* = 0.821) in the mean body mass index of TB-parasitic co-infected cases (mean = 18.1, SD = 1.3) and TB positive cases without parasitic co-infection (mean = 18.2, SD = 2.1). The BMI of co-infected cases didn’t differ significantly from those of non-co-infected cases at different age groups (*χ*^2^ = 4.601, df = 3 and *P* = 0.203) and sex (*χ*^2^ = 0.527, df = 1 and *p* = 0.468).

Among smear positive TB cases whose MUAC were measured, 72.8% (205/281) of them were undernourished and was not significantly associated with gender (*χ*^2^ = 0.831, df = 1 and *P* = 0.362). When MUAC was used to determine nutritional status, 6.4% of the undernourished TB cases and 2.1% of the normal nourished TB cases had parasitic co-infection showing non-significant association (*χ*^2^ = 0.056, df = 1 and *P* = 0.813). As a whole, significant difference was not observed between TB parasitosis co-infections and nutritional status of the TB cases (Table 3).

**Table 3:** Nutritional status and parasitic co-infection of tuberculosis cases in Oromia Special Zone and South Wollo, northeastern Ethiopia, April 2015 to January 2017

	Nutritional status using MUAC			Nutritional status using BMI		*P*-value
	Normal *n* (%)	Under-nourished *n* (%)	*P*-value	Normal *n* (%)	Under-nourished *n* (%)	
Sex						
Males	38 (24.8)	115 (75.2)	0.362	57 (43.8)	73 (56.2)	0.390
Females	38 (29.7)	90 (70.3)		41 (38.3)	66 (61.7)	
Co-infection status						
Co-infected	6 (25.0)	18 (75.0)	0.813	8 (30.8)	18 (69.2)	0.246
Not co-infected	70 (27.2)	187 (72.8)		90 (42.7)	121 (57.3)	

*MUAC* Mid-upper arm circumference, *BMI* Body mass index.

Considering our matched criteria set in the methodology part, 86 participants (55 males and 31 females) were used as control groups. The difference between the nutritional status of smear positive TB cases (mean = 21.4, SD = 2.0) and the control groups (mean = 22.9, SD = 2.5) was statistically significant (*P* = 0. 029) as MUAC parameter was used. However, it is not statistically significant (*P* = 0. 220) when body mass index is considered with average mean (+ SD) of 18.2 (+ 1.95) and 20.7 (+ 2.73) for TB cases and the control groups, respectively.

### *Mycobacterium* culture result

**Fig. 3:**
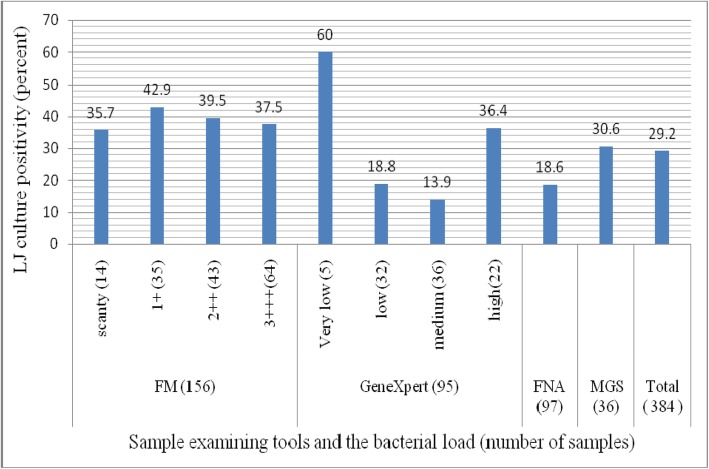
Comparison of results of smear microscopy (FM & FNA) and GeneXpert with bacterial growth on LJ medium, Oromia Special Zone and South Wollo, northeastern Ethiopia, April 2015 to January 2017. FM: Fluorescent microscope. FNA: Fine-needle aspirates. MGS: Missed grading scale. EPTB: Extra-pulmonary tuberculosis. LNs: Lymphadenitis

Growth was observed in 29.2% (*n* = 112) samples after culturing all the smear and GeneXpert positive samples on LJ media. Culture positivity was not significantly different as compared to bacterial load under laboratory examination of smear test in both fluorescent microscope (FM; *P* = 0.455) and GeneXpert (*P* = 0.427). Thirty six samples had missed grading scale (MGS) of the bacterial load (Fig. 3).

Among the EPTB cases, site of infection was detected in 85 participants by the pathologist and TB lymphadenitis (TBLNs) was the most prevalent 85.9% (73/85). Of these TBLNs cervical adenopathy 75.3% (55/73) was the most frequently occurring disease. Of the suspected smear positive EPTB samples, 18.4% were culture positive (Table 4).

**Table 4:** Site of extra-pulmonary tuberculosis infection and the number of culture positives from BHDL, Oromia Special Zone and South Wollo, northeastern Ethiopia, April 2015 to January 2017

Site of EPTB infections	Number of infected cases (%)	Number of culture positives (%)
Cervical LNs	55 (56.1)	8 (14.5)
Axilary LNs	8 (8.2)	2 (25.0)
Supraclavicular LNs	6 (6.1)	1 (16.7)
Other LNs^a^	4 (4.0)	–
Non LN infections	12 (12.2)	4 (33.3)
Not sited (missed)	13 (13.3)	3 (23.1)
Total	98 (100.0)	18 (18.4)

^a^Inguinal, Submandibular and Anterior neck LNs

*BHDL* Bikat Higher Diagnostic Laboratories, *EPTB* Extra-pulmonary tuberculosis, *LNs* Lymphadenitis.

## Discussion

In this study, it has been observed that as family size increased the risk of TB transmission in the community also increased. This might be due to the high risk of contact. Higher rate of infection in farmers and those who cannot read and write might be due to lack of awareness about the disease prevention, immediate diagnosis and treatment [13, 14].

Prevalence of *Mtb* parasitic co-infection in this study (10.8%) is less than the recent co-infection rate (22%) reported from Addis Ababa [32]. This is also true with the higher *Mtb* intestinal parasitic co-infection prevalence rate reported from other parts of Ethiopia in Gondar (33.3%) and Arba Minch (26.3%) [13, 14]. Such greater co-infection rate in other study sites of Ethiopia as compared to the recent finding might be due to the difference in geographical settings and the study participants. The age group of the study participants also vary in that only those whose age were 18 years and older were considered in this study whereas in the case of the former studies those who were younger than 18 were included. Studies in the country revealed that lower age groups possess higher parasitic infection than the older age groups [33]. As there is no gold standard test for parasitic stool examination, differences in the testing mechanisms (direct saline, SAF and Kato-Katz) might also be another factor for the variations of TB parasitic co-infection since the sensitivity of one diagnostic method is different from the other [34].

Similar to this study, findings in Arba Minch showed that the prevalence of intestinal helminth infections was greater (24.4%) than that of intestinal protozoa (6.1%) [14]. Comparable studies toward *Mtb* parasitic co-infection in northwestern Ethiopia also found 44.5 and 7.6% as the proportion of helminthic and protozoa infection, respectively [13]. Although helminthic infection is high in both of the study cases, their percentage varies as compared to this study which might be due to the difference in study subjects as well as the study sites. Contrary to the overall high prevalence of *A. lumbericoides* infection in the country, our finding showed *S. mansoni* to be the most widespread helminthic infections followed by *A. lumbericoides*. This is considered as due to high prevalence and endemicity of schistosomiasis in Oromia Special Zone specifically in Bati and Kemise areas of the study site [35, 36].

Although there was no significant difference in parasitic co-infections among male and female study participants, males were infected in a greater proportion than females similar to the former finding in northwest Ethiopia [13]. By contrast, a report from China showed that females are 2.05 times more likely to acquire IPIs than males [37]. Such differences between Ethiopia and China might be due larger number of males than females in the former and the reverse in the later country.

Several conditions including undernutrition, smoking, *Diabetes mellitus* and co-infections are important risk factors that aggravate the progress of TB [15, 16]. To these effects, our assessments of nutritional status among TB cases using BMI showed about half of the cases were under chronic energy deficiency. Similar studies in other two areas of Ethiopia (Addis Ababa: 39.7% and Arba Minch: 33.3%) found lower percentages as compared to this finding which might be due to the difference in time of taking the measurements [15, 38]. In this study, measurements were taken on the spot of TB screening period when the patients’ body mass is expected to be low. However, the measurements of body mass were taken during the follow up of drug therapy both in Addis Ababa and Arba Minch study cases when their body mass might be improved due to the follow up treatments. In fact, studies in Ghana (51%) and urban Tanzania (56.8%) reported closer prevalence of undernutrition to this finding in *Mtb* parasitic co-infected patients. In all indicated studies, mild malnutrition was at its highest peak than moderate and severe malnutrition [38–40].

The higher percentage (58.6%) of undernutrition in *Mtb* parasitic co-infected cases in this study than parasite free TB cases (41.4%) could imply that co-infection had an impact on the nutritional status of TB cases. Measurements using MUAC also confirmed the findings of BMI in similar way that greater percentage of the TB cases with parasitic co-infection were undernourished than those of non-coinfected cases. Such greater rate of undernourishment in co-infected cases might be due to the parasitic impact. Similar finding of nutritional assessment using both BMI and MUAC parameter suggests co-infection have an impact on the nutritional status of TB cases [32, 41]. In addition, implication of nutritional status difference among TB cases and the control group using both BMI and MUAC may be attributable to tuberculosis. This weight loss agrees with the clinical symptoms criteria for screening tuberculosis cases [24].

The extent of EPTB in Ethiopia is much higher (33%) than the global average (15%) [24]. This finding (25.3%) also agrees with the report which might be due to diagnostic challenges including shortage of pathologists to identify and treat the cases on time in most health institutions. There was no any pathologist to diagnose EPTB in all governmental health institutes including the referral hospital where this study was done. Because of this, all the suspected cases were referred to a single private diagnostic laboratory (BHDL) and remained as the diagnostic challenge of the area.

The overall LJ culture positivity was confirmed in 29.2% (112/384) which is close to the study in Addis Ababa (32.2%, *n* = 124) [42]. On the contrary, the culture positivity of this study is much less than many studies whose findings were 95.9% (118/123), 87.9% (297/338), 53.5% (46/86) and 69.2% (63/91), respectively [43–46]. The less proportion of this study might be due to the delay of culturing time, electric interruption when the specimens were preserved in the refrigerator at the study sites and long distance travel from temporarily stored collection site to ALIPB where the specimens were cultured. These factors increase the chance of bacterial death in the collected sputum samples. Perhaps, it could also be our expectation that bacterial culture positivity might decrease as the sample stayed in the refrigerator for a long duration than immediate culturing.

Regarding both pulmonary and EPTB culturing results, of 286 pulmonary and 98 extra-pulmonary smear positive specimens, 94 (32.8%) and 18 (18.4%) were culture positive, respectively. This proportion is less than that of a study in India who found 329 (61.5%) and 19 (29.2%) as culture positive for *Mtb* in both pulmonary and EPTB cases in that order [47]. A study from different study areas in Ethiopia also reported greater culture positivity of both clinically manifested smear positive pulmonary (79%, 756/953) and extra-pulmonary (38%, 456/1198) TB [24]. Similar studies in northwestern Ethiopia and Addis Ababa showed culture positivity of EPTB as 29.8 and 32.5%, respectively [48, 49]. In all instances, there is less culture positivity of EPTB than pulmonary ones which might be due to cytological suspicion of the specimen by pathologist unlike the detection of disease causative organism itself as in the case of PTB. Moreover, the suspected *Mycobacteria* cellular infection could be paucibacillary which decrease the sensitivity of diagnostic test in EPTB.

The EPTB has different manifestations based on the organs to be attacked and its intent of dissemination in the body. Similar to other relevant studies in the country, this finding also revealed that lymph nodes as the leading organs affected. In fact, the percentage of their infection rate differs as cervical, auxiliary, inguinal, supra-clavicular, sub-mandibular and anterior neck lymph nodes [24, 49]. This higher infection of lymph node is similar to the study reported from Germany [50]. On the other hand, the most common sites involved were the bone/joints and lymph nodes in United States of America [51], whereas the genitourinary system and skin were the common sites of infection reported from Hong Kong [52]. These differences may be attributable to either host or pathogen related factors as well as access to patient sample collection in the clinical settings.

Cervical lymph nodes were found as the most frequently infected anatomical site similar to other report from Ethiopia [53]. Such higher infection rate might be due to the physical proximity of lymph node to the route of infection where the bacilli can easily be picked up by macrophages or dendertic cells that facilitate the transportation of the bacilli in the cervical lymph nodes causing pathology. It could also be expected that the bacteria could easily spread from intra-thoracic lymphatics to the cervical areas. Limitations of this study include examination of a single faecal specimen, that faecal examination was not done for 32.5% of TB patients, that no sputum and faecal examination was done for the control groups and that a small number of participants fulfilled our matched criteria. All these might have a potential bias for the study and should be taken into consideration for future studies.

## Conclusions

In the present study, there is relatively higher helminthic-TB co-infection than those of protozoa parasites. Most of the TB cases in the study were found as undernourished. The prevalence of extra-pulmonary tuberculosis was found as in a greater proportion with cervical lymphadenitis as the principal infection site. Thus, parasitic co-infections and nutritional status of tuberculosis cases should be further studied by treating the parasites and improving nutritional status of the co-infected patients.

## Supplementary information

**Additional file 1.** Multilingual abstracts in the five official working languages of the United Nations

## Data Availability

The datasets supporting the conclusions of this article are included within the article. All data generated or analyzed during this study are included in this published article.

## Abbreviations

AFB: Acid fast bacillus
ALIPB: Aklilu Lemma Institute of Pathobiology;
BHDL: Bikat Higher Diagnostic Laboratories
BMH: Boru Meda Hospital
BMI: Body mass index
DHC: Dessie Health Center
DRH: Dessie Referral Hospital
EPTB: Extra-pulmonary tuberculosis
FM: Fluorescent microscope
FNAs: Fine-needle aspirates
IPIs: Intestinal parasitic infections
LJ: Lowenstein-Jensen
LNs: Lymphadenitis
*Mtb*: *Mycobacterium tuberculosis*
MUAC: Mid-upper Arm Circumference
PTB: Pulmonary tuberculosis
SAF: Sodium acetate-acetic acid-formalin
TB: Tuberculosis
TBLNs: TB lymphadenitis
