# Supplementary material for: High helminthic co-infection in tuberculosis patients with undernutritional status in northeastern Ethiopia
Source: Infect Dis Poverty. 2019 Oct 18;8:88. doi: 10.1186/s40249-019-0600-2 (PMC6798427; doi:10.1186/s40249-019-0600-2)
Supplement: Supplementary file 1 — Additional file 1. Multilingual abstracts in the five official working languages of the United Nations [file 40249_2019_600_MOESM1_ESM.pdf]

Translation of the abstract into the five official working languages of the United Nations

ارتفاع العدوى بالديدان الطفيلية المرافقة لمرضى السل الذين يعانون من حالة نقص التغذية في شمال شرق إثيوبيا

فيكرو غاشاو ، صامويل بيكيلي ، يالمتسيهاي ميكونين ، جيرماي ميدين ، جوبينا أميني ، برهانو إركو

ملخص

الخلفية: يعتبر السل والطفيليات من الأمراض المنتشرة على نطاق واسع في إثيوبيا ، والسبب الرئيسي للوفيات والأمراض، تباعا. لم تكن هناك معلومات عن حالة الإصابات بمرض السل المرافقة بالطفيليات في منطقة أوروميا من اقليم أمهرا وجنوب وولو في إثيوبيا. لذلك تركز هذه الدراسة في المقام الأول على تحديد حالة العدوى بالسل المرافقة بالطفيليات والعوامل المرتبطة بها.

الأساليب: أجريت الدراسة في منطقة أوروميا الخاصة التابعة لولاية أمهرة الإقليمية ومنطقة جنوب وولو، شمال شرق إثيوبيا من أبريل 2015 إلى يناير 2017. مثّلت حالات السل التي أكّدها العاملون في القطاع الصحي بالمؤسسات الطبيةمصدر عيّنة الدراسة. في دراسة تقاطعية تم استقدام 384 من حالات السل الرئوي إيجابي اللطاخة والسل خارج الرئتين. تم فحص عينات البراز التي قدمها المشاركون في الدراسة بحثًا عن العدوى الطفيلية باستخدام الاختبار المجهري الملحي المباشر وتقنية كاتو-كاتز وتقنيات التركيز. تم تحديد الحالة التغذوية باستخدام مؤشر كتلة الجسم ومحيط منتصف العضد. تم تحليل المعطيات باستخدام الطرق الإحصائية الوصفية واختبار مربع كاي لبيرسون.

النتائج: كان معدل انتشار الإصابة بالسل المرافقة بالطفيليات 10.8 % ووصلت نسبة الديدان المعوية إلى 9.7% في حين بلغت نسبة البروتوزوا المعوية 1.9 %. بلغت الحالات المصابة بعدوى طفيلية وحيدة 89.3 % بين الأفراد المصابين. لم ترتبط العدوى المرافقة للوبائين ارتباطا شديدا بالجنس أو بالعمر (الاحتمال (P)أكثر من 0.05) كان معدل انتشار نقص التغذية 58.6 % عند استخدام مؤشر كتلة الجسم و 73.0 %عند استخدام محيط الذراع العلوي مع عدم وجود ارتباط كبير بالجنس. من بين جميع أشكال حالات السل (384) التي تم فحصها للدراسة، كانت النتائج الإيجابية في اختبار البكتيريا نسبيًا أكثر شيوعًا عند الذكور (55.5٪%) مقارنة بالإناث (44.5%). تبين أن التهاب الغدد اللمفاوية في مرض السل (85.9%) هو الشكل الأكثر انتشارًا من مرض السل خارج الرئتين في حين كان تضخم العقد اللمفية الرقبية هو المرض الموجود الأكثر شيوعا (75.3%).

الاستنتاجات: معدل الإصابة المرافقة بالديدان شديد الارتفاع مقارنة بالبروتوزوا المعوية.كانت العدوى الطفيلية المرافقة المنفردة أكثر شيوعًا من العدوى المزدوجة أو المتعددة. كشفت معايير القياسات البشرية في كل من مؤشر كتلة الجسم ومحيط منتصف العضد خطورة أكبر في التعرض لنقص التغذية عند مرضى السل. وبالتالي، ينبغي إخضاع الفحص والعلاج الفوري للطفيليات في مرضى السل والدعم للمكملات الغذائية لمرضى السل المصابين بسوء التغذية، لمزيد من الدراسة مما قد يحسن علاج المرض ويقلل من خطر تعقّده.

Translated from English version into Arabic by Aghilas Mihoub, revised by Arfaoui Firas, through

TRANSLATORS
WITHOUT BORDERS

埃塞俄比亚东北部营养不良结核患者高发寄生虫感染

Fikru Gashaw, Samuel Bekele, Yalemtsehay Mekonnen, Girmay Medhin, Gobena Ameni, Berhanu Erko

摘要

引言：结核病和寄生虫病是埃塞俄比亚广泛流行的疾病，也是造成死亡率和发病率的主要原因。目前尚无关于埃塞俄比亚 Amhara 州 Oromia 特区和南部 Wollo 地区结核病和寄生虫病合并感染情况的资料。因此，本研究主要关注结核病和寄生虫病合并感染的现状及其相关因素。

方法：本研究于 2015 年 4 月至 2017 年 1 月在埃塞俄比亚东北部 Amhara 州 Oromia 特区和南部 Wollo 区进行。研究对象是由卫生机构的医务人员确认的结核病例。在横断面研究中，共收集了

384 例涂阳肺结核和肺外结核病例。对参与者提供的粪便标本，采用直接生理盐水显微镜检查、Kato-Katz 法和浓集法检测寄生虫感染。采用体质指数和中上臂围来确定营养状况。使用描述性统计方法和 Pearson 卡方检验进行数据分析。
结果：结核与寄生虫合并感染率为 10.8%，肠道蠕虫占 9.7%，肠道原虫占 1.9%。合并感染病例中，单发寄生虫感染占 89.3%。两种疾病合并感染与性别、年龄无显著相关性(P > 0.05)。采用体质指数和中上臂围确定的营养不良发生率分别为 58.6%和 73.0%，且与性别无关。本研究的所有结核病例中(384 例)，男性细菌阳性率(55.5%)高于女性(44.5%)。结核性淋巴结炎是最常见的肺外结核(85.9%)，其中宫颈腺病(75.3%)是最常见的疾病。
结论：蠕虫合并感染的发生率明显高于肠道原虫。单发寄生虫合并感染较双重或多重合并感染更为常见。体质指数和中上臂围等相关参数均显示结核患者营养不良的风险较高。因此，应进一步研究结核患者的寄生虫筛查和及时治疗，以及对营养不良者进行营养补充对这类合并感染的疗效，这可能会促进疾病的治疗，并将其复杂性的风险降至最低。

Translated from English version into Chinese by Xin-Yu Feng, edited by Pin Yang

Un nombre élevé de co-infections par helminthes chez des patients tuberculeux en état de dénutrition dans le nord-est de l'Éthiopie

Fikru Gashaw, Samuel Bekele, Yalemtsehay Mekonnen, Girmay Medhin, Gobena Ameni, Berhanu Erko

Résumé

Contexte: La tuberculose et la parasitose sont des maladies largement répandues en Éthiopie et qui sont la principale cause de mortalité et de morbidité, respectivement. Il n'y a aucune information disponible sur les co-infections par la tuberculose et la parasitose dans la zone d'Oromia de la région d'Amhara et dans le sud de Wollo, en Éthiopie. Par conséquent, cette étude s'efforce principalement d'identifier les co-infections par la tuberculose, la parasitose et facteurs associés.
Méthodes: L'étude a été menée dans la zone spéciale d'Oromia de la région d'Amhara et dans la zone du sud de Wollo, au nord-est de l'Éthiopie, d'avril 2015 à janvier 2017. Les échantillons tuberculeux, comme confirmé par le personnel d'établissements de santé, ont été utilisés dans le cadre de cette l'étude. Dans une étude transversale, 384 cas de frottis pulmonaire et extra-pulmonaire positifs pour la tuberculose ont été sélectionnés. Les échantillons fécaux fournis par les participants de l'étude ont été examinés pour rechercher des co-infections parasitaires à l'aide de tests directs microscopiques au sérum physiologique, de la technique Kato-Katz et de techniques de concentration. L'état nutritionnel a été déterminé à l'aide de l'indice de masse corporelle et de la circonférence du milieu du bras. Les données ont été analysées par des méthodes de statistique descriptive et khi-carré Pearson.
Résultats: La prévalence de co-infection tuberculose-parasitose était de 10,8% et la proportion des helminthes intestinaux représentait 9,7% et celle des protozoaires intestinaux, 1,9%. Les cas d'infection parasitaire unique étaient de 89,3% chez les personnes co-infectées. La co-infection des deux maladies n'était pas significativement associée au sexe et à l'âge (P > 0,05). La prévalence de la dénutrition était de 58,6% selon l'indice de masse corporelle et de 73,0% selon la circonférence du bras, sans association significative avec le sexe. Parmi tous les cas de tuberculose (384) sélectionnées pour l'étude, les tests positifs aux bactéries étaient relativement plus fréquents chez les hommes (55,5%) que chez les femmes

(44,5%). La lymphadénite tuberculeuse s'est révélée être la forme de tuberculose extra-pulmonaire la plus répandue (85,9%), l'adénopathie cervicale étant la maladie couramment existante (75,3%).
Conclusions: Le taux de co-infection helminthique était notablement plus élevé que le taux de co-infection par protozoaires intestinaux. Les co-infections parasitaires uniques étaient plus fréquentes que les co-infections doubles ou multiples. L'indice de masse corporelle ainsi que la mesure de circonférence du milieu du bras ont montré que les patients atteints de tuberculose étaient plus à risque de malnutrition. Par conséquent, le dépistage et le traitement rapide des parasites chez les patients tuberculeux, ainsi qu'un apport nutritionnel supplémentaire chez les patients mal nourris devraient être étudié plus en profondeur, afin de potentiellement améliorer le traitement de la maladie et minimiser les risques de complication.

Translated from English version into French by Jurist AKG, revised by Melissa Montrose, through

TRANSLATORS WITHOUT BORDERS

Высокая гельминтозная коинфекция у больных туберкулезом с недостаточным питанием в северо-восточной Эфиопии

Фикру Гашоу, Самуэль Бекеле, Ялемцехай Меконнен, Гирмай Медхин, Гобена Амени, Берхану Эрко

Аннотация
Справочная информация: Туберкулез и паразитоз - широко распространенные заболевания в Эфиопии, которые являются основной причиной смертности и заболеваемости соответственно. Информация о состоянии сочетанной инфекции туберкулеза и паразитоза в зоне Оромия в районе Амхара и Южном Волло (Эфиопия) отсутствует. Таким образом, основное внимание в настоящем исследовании уделяется определению статуса коинфекций туберкулеза и паразитоза и связанных с ними факторов.
Методы: Исследование проводилось в специальной зоне Оромия регионального штата Амхара и зоне Южного Волло на северо-востоке Эфиопии с апреля 2015 года по январь 2017 года. Источником информации для исследования послужили случаи заболевания туберкулезом, подтвержденные медицинским персоналом медицинских учреждений. В перекрестном исследовании было выявлено 384 случая туберкулеза легких и внелегочного туберкулеза с положительным результатом анализа мазка мокроты. Представленные участниками исследования образцы фекалий были исследованы на наличие коинфекций, вызванных паразитами, с использованием методов прямого микроскопического исследования, Като-Катца и концентрации. Состояние питания определялось по индексу массы тела и окружности средней части руки. Анализ данных проводился описательными статистическими методами и методом хи-квадрата Пирсона.
Результаты: Распространенность сочетанной инфекции туберкулеза и паразитоза составила 10,8%, кишечных гельминтов - 9,7%, кишечных простейших - 1,9%. Случаи одиночной паразитарной инфекции среди ко-инфицированных составили 89,3%. Коинфекция обеих болезней не была в значительной степени связана с полом и возрастом ($P > 0{,}05$).

Распространенность недоедания составляла 58,6% по индексу массы тела и 73,0% по окружности средней части руки без какой-либо значимой связи с полом. Среди всех форм туберкулеза (384), отобранных для исследования, бактериальная позитивность была относительно более распространена среди мужчин (55,5%), чем среди женщин (44,5%). Наиболее распространенной формой внелегочного туберкулеза (85,9%) оказался туберкулезный лимфаденит, широко распространенным заболеванием при котором является шейная аденопатия (75,3%).
Выводы: Уровень гельминтной коинфекции в основном выше, чем у кишечных простейших. Одиночная паразитарная коинфекция встречалась чаще, чем двойная или множественная коинфекция. Антропометрические параметры индекса массы тела и окружности средней части руки выявили повышенный риск недоедания у больных туберкулезом. Таким образом, скрининг и оперативное лечение паразитов у больных туберкулезом и поддержка пищевых добавок для недоедающих больных туберкулезом должны быть дополнительно изучены, что может улучшить лечение болезни и минимизировать риск ее сложности.

Translated from English version into Russian by Jurist AKG, revised by Michael Orlov, through

Co-infección, por helmintos altos, en pacientes con tuberculosis con estado nutricional bajo, en el noreste de Etiopía

Fikru Gashaw, Samuel Bekele, Yalemtsehay Mekonnen, Girmay Medhin, Gobena Ameni, Berhanu Erko

Resumen

Antecedentes: La Tuberculosis y la parasitosis son las enfermedades de distribución generalizada, en Etiopía, con la principal causa de mortalidad y morbilidad, respectivamente. No ha existido información sobre la situación de coinfecciones de tuberculosis y parasitosis, en la zona de Oromia , en la región de Amhara y en el sur de Wollo (Etiopía). Por lo tanto, este estudio se centra, principalmente, en determinar la situación de las coinfecciones de tuberculosis y parasitosis y los factores asociados.
Métodos: El estudio se realizó, en la zona especial de Oromia, en el Estado Regional de Amhara y en la Zona del valle del sur, al noreste de Etiopía desde Abril 2015 to Enero de 2017. Los casos de Tuberculosis confirmados, por el personal sanitario de las instituciones sanitarias, fueron la fuente de la muestra del estudio. En un estudio transversal se reclutaron 38 casos positivos de ampollas pulmonares y tuberculosis extra-pulmonar. Se examinaron muestras fecales que proporcionaron los participantes, en el estudio, para detectar infecciones por Co-infección parasitaria, utilizando pruebas salino microscópicas directas, Kato-Katz y técnicas de concentración. El estado nutricional se determinó utilizando el índice de masa corporal y las circunferencias de la parte media del brazo. Los datos se analizaron utilizando métodos estadísticos descriptivos y Pearson chi-square.
Resultados: La prevalencia de coinfección por tuberculosis y parasitosis fue del 10,8% y la proporción de helmintos intestinales representó el 9,7%, mientras que los protozoos intestinales representaron el 1,9%. Los casos de infección parasitaria única fueron de 89,3%, entre las personas co-infectadas. La coinfección de ambas enfermedades no se asoció significativamente con el sexo y la edad ($P > 0.05$). La

prevalencia de desnutrición fue del 58,6%, según el índice de masa corporal y del 73,0% según la circunferencia de la parte media superior del brazo, sin relación significativa con el sexo. Entre todos los tipos de casos de tuberculosis (384) que se examinaron para el estudio, el grupo de la positividad fue relativamente más común en hombres (55.5%) que en mujeres (44.5%). Se encontró que la linfadenitis tuberculosa es la forma más prevalente (85,9%) de tuberculosis extrapulmonar, siendo la enfermedad comúnmente existente la adenopatía cervical (75,3%).

Conclusiones: La tasa de coinfección helmíntica es predominantemente más alta que la protozoo.La coinfección parasitaria única fue más común que la coinfección doble o múltiple. Los parámetros antropométricos del índice de masa corporal y de la circunferencia del brazo medio superior revelaron un mayor riesgo de desnutrición en pacientes con tuberculosis. Por lo tanto, la detección y el tratamiento oportuno de los parásitos, en pacientes con tuberculosis y un soporte de suplementos nutricionales, para pacientes con tuberculosis malnutrida deben estudiarse más a fondo, lo que podría mejorar el tratamiento de la enfermedad y minimizar el riesgo de su complejidad.

Translated from English version into Spanish by Jurist AKG, revised by María Luz Puerta, through

TRANSLATORS
WITHOUT BORDERS
